# Faith, Culture, and Choice: Unraveling the Determinants of Modern Contraceptive Use Among Married Women in Garbatula Sub‐County, Kenya

**DOI:** 10.1155/bmri/8308911

**Published:** 2026-01-22

**Authors:** Mohamed Shukri Elmi, Louisa Ndunyu, Collins Otieno Asweto, Victor Okoth Saoke

**Affiliations:** ^1^ Department of Public Health, Maseno University, Kisumu, Kenya, maseno.ac.ke; ^2^ Department of Community Health, University of Embu, Embu, Kenya, embuni.ac.ke; ^3^ Department of Education, University of Embu, Embu, Kenya, embuni.ac.ke

## Abstract

This study examined the influence of cultural, religious, and gender‐related factors on modern contraceptive use among married women in Garbatula sub‐county, Isiolo County, Kenya. Using a cross‐sectional design, data were collected from 300 married women aged 15–49 years through multistage random sampling. Data were analyzed using descriptive statistics, chi‐square tests, and binary logistic regression. Findings showed that reduced religious influence (AOR = 13.918; 95*%*CI : 2.54–76.30), male involvement in reproductive decision‐making (AOR = 7.765; 95*%*CI : 2.30–26.23), and higher women′s empowerment (AOR = 4.322) were strong positive predictors of modern contraceptive use, whereas rigid cultural norms and patriarchal attitudes were significant barriers. The study concludes that contraceptive behavior in pastoralist settings is shaped by religious and gender norms rather than access alone. It recommends engaging religious leaders, strengthening male‐centered family planning education, and integrating gender‐sensitive strategies within the County Integrated Development Plan (CIDP) to enhance uptake.

## 1. Introduction

Access to contemporary contraceptive techniques is essential for achieving reproductive health, minimizing unwanted pregnancies, improving mother and child health outcomes, and empowering women worldwide [1]. Modern contraceptives allow couples and individuals to manage their fertility, space births, and reach desired family sizes, directly contributing to national development goals. Despite these acknowledged benefits, the use of contemporary contraceptive techniques is inconsistent in many parts of sub‐Saharan Africa, affected by a variety of social, cultural, and religious variables [2].

Cultural and religious attitudes are commonly regarded as significant predictors of contraceptive practice. Many African civilizations base reproductive decisions on religious teachings and cultural expectations that encourage high fecundity, male dominance, and distrust of modern family planning technologies [2, 3]. Cultural norms, such as the expectation for women to have multiple children or to delegate fertility decisions to male partners, frequently hinder women′s reproductive autonomy [4]. Similarly, religious teachings can either discourage or support the use of contemporary contraception, depending on how religious leaders understand and convey doctrines [5].

Several studies have looked at the relationship between cultural, religious, and contraceptive use patterns. For example, Cole and Geist [6] and [7]) found that in environments where religious and cultural limitations are regarded to be less rigid, contraceptive use is much higher. These studies demonstrate that reducing traditional obstacles through education and health promotion leads to increased acceptance and use of family planning options. Similarly, Adedini et al. [5] showed that the active participation of religious leaders and men in reproductive health initiatives can dramatically boost contraceptive acceptance, underlining the importance of shifting societal norms at both the community and family levels. Additionally, Kriel et al. [8] discovered that public support for family planning from religious authorities significantly increased community acceptance in South Africa.

However, other investigations provide mixed findings. Bashir [9] revealed that even where family planning education was available, deep‐seated religious opposition remained to limit contraceptive usage in parts of Nigeria, demonstrating that mere exposure to information is not always sufficient to fight entrenched views. Despite socioeconomic advancements, Kabagenyi et al. [10] found that son preference and cultural beliefs about large family sizes continue to be significant impediments in many African contexts. Low contraceptive use persists despite family planning education, owing to the significant cultural and religious significance of fertility in many African countries. Fertility is frequently viewed not as a personal decision, but as a communal, religious, and societal expectation linked to lineage preservation, social position, and divine will [11]. Even when women are given information on the advantages of family planning, social pressures to comply with cultural norms, such as having big families or having male children, can outweigh individual knowledge or intentions. Religious beliefs that prohibit contraception use, when absorbed by communities, create additional barriers that cannot be overcome through education alone [12]. Thus, without addressing the underlying cultural and religious narratives that drive reproductive behavior, awareness campaigns may have limited impact on real contraceptive uptake, as demonstrated by Sinai et al. [13].

Against this context, this study investigated the cultural and religious factors influencing contemporary contraception use among married women in Garbatula sub‐county. Garbatula is a unique setting with a significant Islamic influence, seminomadic pastoralist lifestyles, and established traditional practices, making it an important site for studying how cultural and religious factors influence reproductive health behaviors [14]. Although previous studies have looked at these interactions in broader Kenyan or African contexts, there is little empirical research that focuses on distant, mostly pastoralist villages like Garbatula. Furthermore, these studies were frequently descriptive and did not thoroughly examine the predictive power of cultural and religious elements using inferential statistical approaches.

This study is aimed at filling this gap by using descriptive and logistic regression analysis to investigate the impact of cultural gender norms, religious beliefs, and leadership support on modern contraception use. As a result, the study provides evidence‐based insights that can be used to build culturally responsive reproductive health interventions and policies. The study emphasizes the vital relevance of religious leader engagement, male participation, and women′s empowerment in moving societal norms toward more positive attitudes regarding family planning. Thus, our study is justified by the need to provide context‐specific knowledge that addresses continuing barriers to contemporary contraceptive usage in rural Kenya and similar sociocultural situations throughout Africa.

This study contributes novel empirical evidence by quantitatively examining how religious, cultural, and gender‐related factors jointly influence contraceptive use among pastoralist Muslim communities in Garbatula sub‐county, an area rarely captured in national or international reproductive health research. Although previous studies in Ethiopia, Uganda, and northern Kenya have qualitatively described religious and cultural barriers, few have applied multivariate quantitative models to measure the magnitude of these effects. By integrating cultural and faith‐based determinants within a logistic regression framework, this study bridges an existing methodological and contextual gap, providing a more nuanced understanding of contraceptive behavior in marginalized pastoralist populations.

### 1.1. Conceptual Framework

**Figure 1:**
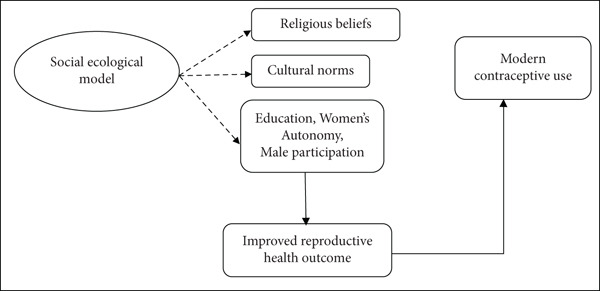
Conceptual framework of the study.

The social ecological model (SEM), which describes how various interacting factors at the individual, interpersonal, community, and societal levels shape individual behaviors, served as the foundation for this investigation as explained by Stavitz [15]. According to this paradigm (Figure 1), a mix of religious, cultural, and personal variables affect married women′s usage of contemporary contraception in Garbatula sub‐county. At the individual level, education and women′s autonomy affect awareness, decision‐making, and perceived control over fertility choices. At the interpersonal level, male dominance and spousal communication influence whether contraceptive discussions and decisions occur within the family. At the community level, cultural acceptance, traditional norms, and religious teachings determine the social approval or disapproval of contraceptive practices. At the societal level, institutional and religious leadership support can either promote or hinder family planning behaviors.

Therefore, the framework makes the assumption that education, women′s autonomy, and male participation mitigate the impacts of religious beliefs, cultural norms, and male domination, which are major predictors of contraceptive use. Modern contraception use is more likely when women are empowered, religious leaders are supportive, and communities are more culturally accepting. These factors ultimately contribute to better reproductive health outcomes.

### 1.2. Hypotheses of the Study

Based on the reviewed literature and the conceptual framework grounded in the SEM, this study was guided by the following hypotheses. The hypotheses were formulated to test the statistical relationship between cultural and religious determinants and the use of modern contraceptives among married women in Garbatula sub‐county, Kenya.

**Hypothesis 1.**

(Null Hypothesis): Religious beliefs, cultural norms, and male dominance have no statistically significant influence on modern contraceptive use among married women in Garbatula sub‐county.

**Hypothesis 2.**

(Alternative Hypothesis): Religious beliefs, cultural norms, and male dominance significantly influence modern contraceptive use among married women in Garbatula sub‐county.

## 2. Methodology

### 2.1. Study Area

This study was carried out in Garbatula sub‐county, Isiolo County, Kenya. Garbatula is largely a pastoralist community, and traditional nomadic practices have a significant impact on the cultural and social dynamics of reproductive health. The area′s contemporary contraceptive prevalence rate is 29%, which is much lower than the national average of 59% [16]. Although over 60% of women in the region have access to family planning services, contraception use remains at 25%. Garbatula sub‐county has approximately 10,000–12,000 houses of varying sizes and compositions. The health facilities in Garbatula sub‐county are classified into levels according to Kenya′s healthcare system framework.

### 2.2. Variable Description

To examine the cultural and religious factors that influence married women in Garbatula sub‐county′s usage of contemporary contraceptives, the study used a structured collection of variables as indicated in Table 1. In order to determine whether respondents now use any contemporary method of contraception, the dependent variable was modern contraceptive use, which was measured dichotomously (0 = No, 1 = Yes). This is a typical measure in research on reproductive health [17].

**Table 1:** The study variables.

**Variable category**	**Variable**	**Measurement**	**Scale**	**Operationalization**
Dependent variable	Modern contraceptive use	Current use of any modern contraceptive method	Dichotomous	0 = No, 1 = Yes
Independent variable	Traditional beliefs	Belief in traditional family planning methods	Dichotomous	0 = No, 1 = Yes
	Cultural acceptance	Community acceptance of modern contraceptives	Categorical	1 = Not accepted, 2 = Somewhat accepted, 3 = Accepted
	Role of elders	Influence of community elders on family planning decisions	Categorical	1 = No influence, 2 = Some influence, 3 = Significant influence
	Type of marriage	Type of marriage (monogamous/polygamous)	Categorical	1 = Monogamous, 2 = Polygamous
	Religious beliefs	Impact of religious beliefs on contraceptive use	Categorical	1 = No impact, 2 = Some impact, 3 = Significant impact

The traditional beliefs as a variable evaluated respondents′ support for conventional family planning techniques. The adoption of contemporary contraception may be discouraged in many conservative communities by traditional beliefs that place a strong emphasis on large family sizes and natural fertility [10]. These ideas can be firmly established in cultural customs, transmitted from one generation to the next, and upheld by social support. According to Alvergne and Stevens [18], these kinds of beliefs are major obstacles to the adoption of modern contraceptives, especially in seminomadic or rural areas where traditional medicine and practices are prevalent. Cultural acceptance, which is a categorical variable, measured how well received the usage of contemporary contraception is in the community. Individual health practices are significantly shaped by societal norms and attitudes [19]. People may refrain from using contraceptives in situations when it is thought to be abnormal or immoral out of fear of social rejection, stigma, or criticism from friends and family [20]. This variable′s inclusion recognizes that health practices are ingrained in larger societal contexts rather than being just personal decisions.

To illustrate the hierarchical structure of decision‐making in many African societies, the influence of community elders was added. Elders, especially male leaders and clan chiefs, frequently act as cultural and moral authorities that set standards for appropriate family behavior, including reproductive decisions [21]. Their endorsement or condemnation might have a big impact on the choices made by younger generations regarding contraception. According to Mochache et al. [22], this variable acknowledges the elders′ function as cultural guardians and influencers of reproductive norms. Marriage as a variable is distinguished between polygamous and monogamous marriages. A woman′s degree of autonomy in making reproductive decisions is influenced by her marital status in addition to her desires for the size of her family [23]. To preserve their position in the family, women in polygamous relationships could feel increased pressure to have more children, which could limit their willingness or capacity to use contraception. On the other hand, monogamous women might have greater negotiating power or room when it comes to fertility planning [24].

This study also evaluated how much religious beliefs affect the usage of contraceptives. Moral frameworks that direct reproductive behavior are frequently provided by religious leaders and institutions [5]. Religion is a significant driver of health behavior since it can either encourage or forbid the use of contemporary family planning techniques in various situations. The doctrinal and interpretive differences that influence attitudes about the use of contraceptives both within and between religious communities are explained by this variable. Although several independent variables, such as cultural acceptance and religious influence, were measured on ordinal scales (1–3 categories), they were treated as categorical variables in the binary logistic regression to simplify interpretation and maintain consistency with previous reproductive health studies [17, 25]. Nevertheless, supplementary analyses using ordinal logistic regression were conducted and produced comparable patterns of association, confirming the robustness of the results.

To account for possible confounding influences, key demographic covariates such as age, level of education, parity, and household income were included in the multivariate model. These variables are known to affect contraceptive use independently and were controlled for to isolate the specific effects of religious and cultural predictors. Including these covariates strengthens the model′s internal validity and reduces the likelihood that observed associations are spurious. The inclusion of these demographic covariates was maintained in subsequent regression models to ensure consistent statistical control across analyses.

### 2.3. Study Design, Sample Strategy, and Sampling Size

The study used a cross‐sectional research approach, outlined by Saoke et al. [26–28], to offer an overview of modern contraception use among married women in Kenya. As a cross‐sectional design, this study identifies associations rather than causal relationships between cultural and religious variables and contraceptive use. This method is especially useful since it enables efficient data collecting in a single timeframe, lowering expenses and logistical issues [27]. The study used a multistage sampling technique with numerous structured phases to choose a representative sample from the fragmented population of Garbatula sub‐county. At first, the sub‐county was organized into administrative units, namely the Garbatula, Kinna, and Sericho wards. A random number generator was used to select a total of 10 clusters. Within these chosen clusters, systematic random sampling was used to choose households. For example, if a cluster had 500 households, every 10th household was chosen starting from a randomly determined location.

Subsequently, all married women aged 15–49 years in each selected home were identified, and if there were numerous eligible women, one was chosen at random using a basic random procedure, such as drawing names. This method helped to reduce logistical constraints and selection bias, improving the study′s diversity and adaptability in the pastoralist context [29]. The sample size for the study was determined using Cochran′s proportional sample size determination formula, which is presented in Equation (1). Cochran′s proportional sample size determination formula is provided by Daraz et al. [30].

(1) n0=z2pqe2,

where *n*_0_ is the sample size for a population with an infinite size, *p* is the fraction of the population that uses contemporary family planning, *z* is the normalized score that reflects a 95% confidence interval (CI), which is equal to 1.96, and *e* is the intended margin of error, which is 5% in this study. The formula takes into account crucial statistical characteristics such as the desired confidence level (usually 95%), acceptable margin of error (e.g., 5%), and anticipated population proportion, making it appropriate for research that wants to generalize their findings to a larger population.

In this study, the population of married women in Garbatula sub‐county is both huge and diversified. Cochran′s formula assures that the chosen sample is appropriately representative, improving the results′ external validity and generalizability. It also accounts for population variability, producing a sample size that compromises accuracy and resource restrictions [31]. The method′s ability to handle varying confidence levels and margins of error makes it ideal for social and public health research, where precision and representativeness are essential. Thus, Cochran′s formula was justified as the best method for determining a scientifically legitimate and statistically sound sample size for this study. All married women aged 15–49 years residing in the selected households for at least 6 months prior to the survey were eligible for participation. Women who were pregnant or reported current infertility were excluded to ensure comparability in contraceptive‐use potential. Out of 320 eligible women approached, 300 completed the questionnaire, yielding a response rate of 93.8%. Nonresponses (6.2%) were primarily due to temporary household absence or refusal. No systematic differences were observed between respondents and nonrespondents in terms of age and marital status, based on community health records. This approach enhanced the representativeness of the sample and reduced nonresponse bias in the final analysis.

### 2.4. Ethical Consideration

To ensure that this human study followed ethical guidelines, relevant institutional review boards were consulted (NACOSTI/P/24/41029 and MUSERC/01349/24), as well as consent from local health authorities and administrative officials. Prior to participant recruitment, community entry protocols were observed through consultations with local leaders, elders, and religious authorities, who acted as cultural gatekeepers to endorse the study and facilitate trust among community members. All participants gave informed consent, and the study′s purpose, procedures, potential hazards, and benefits were thoroughly explained in both English and the local dialect to improve comprehension. Participants were assured that their participation was voluntary and that they could withdraw at any moment without penalty. No financial incentives were provided; however, light refreshments and reimbursement for transport were offered where necessary to acknowledge participants′ time. To minimize potential gender and power imbalances during interviews, particularly on sensitive reproductive health issues, enumerators were gender‐matched with respondents wherever possible, and all research assistants underwent training on cultural sensitivity, confidentiality, and noncoercive communication. To ensure confidentiality, personal identifiers were removed from the data, and no individuals were identified in reports or publications. All procedures adhered to internationally recognized ethical standards, including the principles of the Declaration of Helsinki [32] and the Council for International Organizations of Medical Sciences (CIOMS, [33]) guidelines for research involving human subjects. These frameworks emphasize respect for persons, beneficence, justice, and cultural sensitivity in the protection of participants′ rights and welfare. All shared data and materials were anonymized in compliance with ethical approval requirements from NACOSTI and MUSERC to ensure participant confidentiality.

### 2.5. Data Collection Procedure

A thorough questionnaire was developed, which included demographic information, contraceptive use, and relevant social and cultural factors. The questionnaire contained both closed and open‐ended questions. Before refining the questionnaire, a preliminary test with 30 participants confirmed its clarity. Research assistants fluent in local dialects were recruited and trained on ethical problems and data collection methods. To promote participation, response rates were tracked, and follow‐ups were conducted as needed. Data was then entered into SPSS while remaining confidential and accurate, followed by a comprehensive cleaning procedure. Descriptive and bivariate analyses detailed demographics, whereas logistic regression examined the association between religion, culture, and contraceptive use, yielding statistically significant results (*p* < 0.05). This extensive data collection strategy yielded genuine insights on improving contraceptive access for women in the region.

### 2.6. Statistical Analysis

The statistical procedure in this study employed SPSS to examine the relationships between several religious and cultural variables and the use of contemporary contraception among married women in Garbatula sub‐county, Kenya. Descriptive statistics were then utilized to summarize the individuals′ religious and cultural data. This included central tendency measures (mean and median) and frequency distributions for categorical variables.

Additionally, bivariate analyses, such as chi‐square tests, were used to investigate the links between individual religious, cultural characteristics, and contraception use. These tests assisted in determining whether the percentage of contraceptive use differed significantly across levels of the respective variables. A binary logistic regression analysis was then carried out to examine the independent effects of various factors on the likelihood of taking modern contraception. This technique was adopted because it considers the binary nature of the dependent variable (current use of modern contraception: Yes/No) and allows for the management of confounding variables [34]. Equation (2) describes the binary regression modeling.

(2) Y=β0+β1X1+β2X2+β3X3+β4X4⋯..βn+μ,

where *Y* is the use of modern contraceptives, *β*_0_ is the intercept, *β*_1_, *β*_2_, *β*_3_, *β*_4_, and *β*_5_ are the regression coefficients of independent variables, X_1_ is the religious factors, X_2_ is the cultural factors, *β*_n_ is other variables, and *μ* is the error term.

Diagnostic procedures were used to assess data quality and fitness, including the Cronbach test for consistency [35]. Similar models suitable for this type of investigation are ordinary least squares (OLS), system generalized method of moments (GMM), and panel vector autoregression (VAR). However, this study incorporated several predictor variables. As a result, the study required a statistical method to identify various relationships between several explanatory variables and a single continuous outcome. As a result, the study utilized binary logistic regression as our major analytical model. Binary regression was also chosen over other models because it evaluates the study′s predictor variables concurrently rather than sequentially [25]. Cronbach′s alpha was used to evaluate the instrument′s reliability; the result was 0.812, which showed strong internal consistency among the items measuring cultural and religious components. Data were checked for distributional irregularities and missing values before inferential analysis. Less than 3% of all observations were missing data, which were removed using listwise deletion because Little′s MCAR test showed that the missing data pattern was random (*p* > 0.05).

The robustness of the regression results was assessed using model diagnostics. The Hosmer–Lemeshow goodness‐of‐fit test showed that the model fit the data well (*χ* ^2^ = 7.83, *p* = 0.45). Variance inflation factors (VIFs), which were used to analyze multicollinearity, were all less than 2.0, indicating that the predictors were independent. Furthermore, the model explained almost 39% of the variation in the use of contemporary contraceptives, as indicated by the Nagelkerke R^2^ value of 0.392. The adequacy of the chosen analytical procedures was validated by the satisfaction of all chi‐square test assumptions, including independence of observations and predicted cell counts more than five in more than 80% of cases. These diagnostic checks confirmed the statistical adequacy of the models, providing a reliable basis for interpretation of the results presented in this study.

The analytical process was thoroughly described to encourage reproducibility and transparency. According to their operational definitions, independent and dependent variables were coded (see Table 1). Categorical variables were dummy‐coded for the binary logistic regression, and the following reference categories were assigned: cultural flexibility (restrictive = reference), women′s empowerment (low = reference), male involvement (low = reference), and religious impact (high = reference). Odds ratios with relation to the most conservative or restricted category for each predictor might be meaningfully interpreted owing to this arrangement. To allow for independent verification, the SPSS syntax used for all bivariate, multivariate, and descriptive analyses is included with the supplemental materials.

## 3. Results and Discussion

### 3.1. Demographic Characteristics of Respondents

An overview of the social makeup of married women in Garbatula sub‐county is illustrated in Table 2. The majority (50.83%) were in the reproductive prime, which corresponds with a larger desire for contraception because of child spacing or limiting preferences during this time in life. In line with the wider religious composition of northeastern Kenya, where Islamic teachings may have an impact on reproductive norms and contraceptive behavior [2], Muslims made up the largest religious group (70.43%). The majority of respondents (38.87%) had a certificate or diploma, indicating a moderate degree of educational attainment and probable openness to knowledge about contemporary contraceptives. The low percentage of graduates (8.31%), however, suggests that access to thorough sexual and reproductive health education may be restricted.

**Table 2:** Demographic features of the participants.

	**Frequency**	**%**
Age		
< 25 years	18	5.98
26–35 years	153	50.83
36–49 years	130	43.19
Religion		
Muslim	212	70.43
Catholic	55	18.27
Protestant	32	10.63
No religion	2	0.66
Education level		
Primary and below	75	24.91
Secondary	84	27.91
Certificate/diploma	117	38.87
Graduate and above	25	8.31
Source of Income		
Employed	62	20.60
Pastoralist	147	48.84
Business	63	20.93
Farming	29	9.63
Years lived in Isiolo		
2–5 years	106	35.26
6–10 years	168	55.81
11–15 years	27	8.97

Nearly half (48.84%) were pastoralists, a seminomadic lifestyle that can make it difficult to consistently obtain family planning and other health services and facilities. Furthermore, the majority (55.81%) had been residents in Isiolo for 6–10 years, indicating a comparatively stable population with well‐established social and cultural ties that might support traditional norms influencing the use of contraceptives as indicated by Daraz et al. [30].

When analyzing the use of contraceptives, these demographic parameters offer crucial background. Though this can be tempered by religious teachings and the practical obstacles of pastoralism, younger and moderately educated women are typically more receptive to contraceptive techniques (Mahamed 2023). The necessity for culturally and religiously sensitive family planning programs that involve religious leaders and customize messaging to Islamic principles is highlighted by the large percentage of Muslims. Furthermore, to guarantee consistent access, outreach approaches should be transportable and community‐integrated, according to pastoralist livelihoods. All factors considered, the demographic profile emphasizes how sociocultural identity and health service use interact, highlighting the necessity of focused, situation‐specific interventions [36].

Further analysis revealed that contraceptive use varied notably across key demographic groups. Women aged 26–35 years, representing the peak reproductive period, reported the highest levels of modern contraceptive use compared with those below 25 or above 36 years. Similarly, educational attainment showed a strong positive association with contraceptive uptake; women with a diploma or higher qualifications were more likely to use contraception than those with primary education or below. Employment status also influenced use, as employed women demonstrated greater contraceptive adoption than those engaged in pastoralism, reflecting differences in access to health information and autonomy in reproductive decision‐making. These patterns are consistent with evidence from similar Kenyan and sub‐Saharan contexts [5, 7], confirming that demographic and socioeconomic factors interact with cultural and religious determinants to shape contraceptive behavior.

### 3.2. The Chi‐Square Test of Independence

The chi‐square analysis in Table 3 was employed to determine whether there is a significant association between two categorical variables. In this study it checks whether cultural and religious factors are statistically associated with modern contraceptive use (Yes/No). This statistical test is used to examine whether there is a significant association between two categorical variables, in this case, religious factors (e.g., beliefs, leader support, and perception of children) and modern contraceptive use (categorized as “Yes” or “No”).

**Table 3:** Association between religious factors and modern contraceptive use.

	**Modern contraceptive use**		**χ**2	**p** **value**
	**No** **n** **(%)**	**Yes** **n** **(%)**		
Religious beliefs play any role in the contraceptive use in the family				
Yes	188 (65.5)	99 (34.5)	5.149	0.023
No	5 (35.7)	9 (64.3)		
Extent that religious beliefs affect contraceptive use				
To a very low extent	4 (44.4)	9 (55.6)	15.063	0.002
To a low extent	35 (52.2)	32 (47.8)		
To a moderate extent	75 (60.5)	49 (39.5)		
To a great extent	79 (78.2)	22 (21.8)		
My religious leader speaks publicly in favor of family planning/child birth spacing				
Disagree	75 (78.9)	20 (21.1)	18.605	< 0.001
Moderately agree	93 (62.0)	57 (38.0)		
Agree	25 (44.6)	31 (55.4)		
My religion supports the use of contraceptives				
Disagree	91 (70.5)	38 (29.5)	11.261	0.004
Moderately agree	87 (64.4)	48 (35.6)		
Agree	15 (40.5)	22 (59.5)		
My religion encourages birth control using modern contraceptives				
Disagree	84 (71.2)	34 (28.8)	9.463	0.009
Moderately agree	94 (63.5)	54 (36.5)		
Agree	15 (42.9)	20 (57.1)		
My religion views children as gifts from God, and they should not be stopped from coming				
Disagree	36 (65.5)	19 (34.5)	4.116	0.249
Moderately agree	87 (64.0)	49 (38.0)		
Agree	70 (63.6)	40 (36.4)		
Highly religious neighbors slow modern contraceptive use				
Disagree	52 (65.8)	27 (34.2)	1.460	0.692
Moderately agree	96 (61.1)	61 (38.9)		
Agree	45 (69.2)	20 (30.8)		

The findings of the chi‐square test showed statistically significant relationships between the usage of contemporary contraceptives by married women in Garbatula sub‐county and several religious characteristics (Table 3). Interestingly, there was a significant correlation between the usage of contraceptives and whether religious beliefs influenced family planning decisions in any way (*χ* ^2^ = 5.149, *p* = 0.023). The limiting influence of religious beliefs on family planning decisions was highlighted by the fact that women who reported no religious influence were more likely to use contemporary contraception (64.3%) than those who admitted a religious role (34.5%). These results are consistent with research that demonstrates how moral teachings and social expectations might influence reproductive behavior through religion [37]. There was also a graded correlation between the degree of religious influence (*p* = 0.002). Contraceptive use was highest among women who claimed minimal religious influence (up to 55.6%) and lowest among those who indicated religion affected them “to a great extent” (21.8%). This gradient implies that opposition to contemporary family planning techniques may be directly correlated with the degree of religious adherence, a pattern seen in comparable research conducted throughout sub‐Saharan Africa [2].

One important predictor was the support of religious leaders: Those who felt that their religious leaders publicly supported family planning were far more likely to use contraceptives (55.4%) than those who disagreed (21.1%) (*χ* ^2^ = 18.605, *p* < 0.001). This research emphasizes how influential religious leadership is in establishing social norms and defending contemporary medical procedures [38]. It also implies that, especially in conservative or faith‐based contexts, religious leaders can be extremely helpful supporters in reproductive health initiatives. Contraceptive usage was also substantially correlated with beliefs regarding whether religion promotes birth control and whether it supports the use of contraceptives (*p* = 0.004 and *p* = 0.009, respectively). In line with previous studies that demonstrate that theological support for family planning increases acceptability and use, women who agreed that their religion supported contraceptives reported the highest utilization (59.5%) [39].

Contrarily, perceptions of neighbors′ religiosity (*p* = 0.692) and the belief that “children are gifts from God and should not be stopped from coming” (*p* = 0.249) did not significantly correlate with the use of contraceptives, indicating that personal interpretations and the influence of religious leaders may be more important in reproductive decision‐making than more general communal attitudes [5]. All factors considered, these results show that religion has a significant impact on contraceptive practices, both as a personal belief system and through institutional voices. In comparable sociocultural contexts, addressing religious concerns and involving church leaders in lobbying could greatly increase the usage of contemporary contraception.

The findings in Table 4 show that cultural attitudes have a major impact on contemporary contraception use among married women in Garbatula sub‐county. Women who disagreed with the statement “men decide when to have a child” were substantially more likely to use contraception (62.5%) than those who somewhat or strongly agreed (*p* < 0.001). This complements the findings of Anwar et al. [40], who found that male‐dominated decision‐making inhibits women′s autonomy in reproductive health choices. Similarly, women who disagreed with the assumption that wives require husbands′ encouragement to use contraception had higher contraceptive usage rates (59.3%), underlining the importance of female agency (*p* = 0.001), which is comparable with the findings by Sarfraz et al. [41].

**Table 4:** Association between cultural factors and modern contraceptive use.

	**Modern contraceptive use**		**χ**2	**p** **value**
	**No** **n** **(%)**	**Yes** **n** **(%)**		
In our culture, men are the ones who decide when to have a child				
Disagree	15 (37.5)	25 (62.5)	15.859	< 0.001
Moderately agree	83 (64.3)	46 (35.7)		
Agree	95 (72.0)	37 (28.0)		
Wives need husbands′ encouragement of modern contraceptive use				
Disagree	11 (40.7)	16 (59.3)	15.158	0.001
Moderately agree	46 (54.1)	39 (45.9)		
Agree	136 (72.0)	53 (28.0)		
In our culture, a woman does what the man wants in fear that he would get another woman				
Disagree	25 (75.8)	8 (24.2)	3.955	0.138
Moderately agree	115 (60.2)	76 (39.8)		
Agree	53 (68.8)	24 (31.2)		
Cultural factors are very important in women′s decisions about family size and contraception				
Disagree	13 (65.0)	7 (35.0)	1.051	0.789
Moderately agree	59 (66.3)	30 (33.7)		
Agree	106 (64.2)	59 (35.8)		
Strongly agree	15 (55.6)	12 (44.4)		
Son preference play the most dominant role to birth control				
Strongly disagree	13 (72.2)	5 (27.8)	2.172	0.704
Disagree	29 (64.4)	16 (35.6)		
Moderately agree	60 (61.2)	38 (38.8)		
Agree	71 (67.6)	34 (32.4)		
Strongly agree	20 (57.1)	15 (42.9)		
Men are involved in programs that seek to address women′s uptake of contraception				
Strongly disagree	27 (77.1)	8 (22.9)	23.786	< 0.001
Disagree	58 (77.3)	17 (22.7)		
Moderately agree	69 (67.0)	34 (33.0)		
Agree	33 (45.2)	40 (54.8)		
Strongly agree	6 (40.0)	9 (60.0)		

However, the relationship between the belief that “women act out of fear that men may find another partner” and contraceptive use was not statistically significant (*p* = 0.138), implying that fear of marital instability may not directly deter contraceptive use in this community, in contrast to the findings of Shultz et al. [42], who observed strong fear‐based fertility behavior in West Africa.

Beliefs about the cultural relevance of family size, as well as son preference, were not substantially connected with contraceptive use. This contradicts research by D’Souza et al. [7] who found that son preference greatly influences contraceptive practice in many African cultures, implying that Garbatula′s opinions may be altered. Women and couples have the option to select economic sustainability, children′s education, and healthcare access over traditional family size and gender preferences. Furthermore, ongoing community sensitization efforts by nongovernmental organizations (NGOs) and government health initiatives may have helped to promote gender equality, family planning education, and the value of smaller, well‐supported families rather than solely male offspring [43]. Thus, current socioeconomic realities, rather than old cultural standards, may now have a greater impact on contraceptive decision‐making in Garbatula sub‐county.

Men who participated in reproductive health programs were more likely to utilize contraception (*p* < 0.001). Women who agreed or strongly agreed that men were involved used contraception at a significantly greater rate. This finding is consistent with Tilahun et al. [44], who stated that male involvement initiatives greatly increase contraceptive uptake. In places like Garbatula, where cultural traditions frequently grant men extensive responsibility over family affairs, including reproductive decisions, their support is critical for successful contraception adoption. When men actively participate in health education campaigns, they learn more about the benefits of family planning, which leads to increased acceptance, fewer misconceptions, and better collaborative decision‐making with their spouses [45].

Furthermore, male engagement frequently promotes improved spousal communication about reproductive objectives, lowering the concerns and hostility traditionally associated with contraception use [46]. As a result, women feel more empowered and supported to use contemporary contraception when their partners participate in family planning programs. Overall, the results we obtained suggest that empowering women and actively engaging males are critical methods for increasing contraceptive use in historically conservative communities.

### 3.3. The Relationship Between Cultural, Religious Factors and Modern Contraceptive Use

Before running the inferential analyses, diagnostic tests were conducted to verify key assumptions for both the chi‐square and logistic regression models. For chi‐square analyses, expected cell frequencies exceeded five in more than 80% of cells, and all observations were independent. Logistic regression assumptions were also satisfied: the dependent variable was binary, predictors were independent, and multicollinearity was minimal (VIF < 2.0). These checks confirmed that the data met the statistical requirements for valid interpretation of the regression coefficients.

Using binary logistic regression in Table 5, this study looked at how cultural and religious characteristics affect modern contraception use among married women in Garbatula sub‐county. The findings indicate that women who stated that religious and cultural views did not influence contraceptive use were more likely to use contraception (adjusted odds ratio [AOR] = 3.704, *p* = 0.074); nevertheless, the adjusted effect was not statistically significant. However, the lower the perceived influence of religious and cultural beliefs, the higher the odds of contraceptive use (AOR = 13.918, *p* = 0.002 for very low extent), which is consistent with the findings of Arousell and Carlbom [37], who found that weaker cultural and religious restrictions are associated with higher contraceptive adoption. Strong cultural and religious standards in many traditional cultures have long discouraged contraception usage, considering it as incompatible with expectations of large families or divine control over fertility [47]. When people view these beliefs to be less binding or persuasive, they are more likely to prioritize personal or health‐related reasons for family planning, such as economic stability, maternal health, and children′s well‐being [48]. This trend in Garbatula could also be attributed to the increased influence of education, exposure to family planning campaigns, and urbanization, all of which frequently challenge traditional conventions and promote more individualized fertility decisions. As women become more aware and empowered, they are less bound by cultural norms and hence more willing to use modern contraception methods.

**Table 5:** The binary logistic regression analysis.

	**Modern contraceptive use** **n** **(%)**	**OR (95% CI for OR)**	**p** **value**	**AOR (95% CI for AOR)**	**p** **value**
Religious and cultural beliefs play any role in the contraceptive use in the family					
Yes	99 (34.5)	Ref			
No	9 (64.3)	3.418 (1.115–10.476)	0.031	3.704 (0.881–15.569)	0.074
Extent that religious and cultural beliefs affect contraceptive use					
To a very low extent	9 (55.6)	4.489 (1.110–18.150)	0.035	13.918 (2.539–76.299)	0.002
To a low extent	32 (47.8)	3.283 (1.675–6.436)	0.001	3.741 (1.634–8.564)	0.002
To a moderate extent	49 (39.5)	2.346 (1.295–4.249)	0.005	2.525 (1.215–5.245)	0.013
To a great extent	22 (21.8)	Ref		Ref	
My religious leader speaks publicly in favor of family planning/child birth spacing					
Disagree	20 (21.1)	Ref		Ref	
Moderately agree	57 (38.0)	2.298 (1.270–4.160)	0.006	2.019 (0.970–4.202)	0.060
Agree	31 (55.4)	4.650 (2.260–9.569)	< 0.001	7.085 (2.929–17.135)	< 0.001
My religion supports the use of contraceptives					
Disagree	38 (29.5)	Ref		Ref	
Moderately agree	48 (35.6)	1.321 (0.788–2.216)	0.291	1.260 (0.653–2.433)	0.491
Agree	22 (59.5)	3.512 (1.646–7.493)	0.001	2.974 (1.165–7.595)	0.023
My religion encourages birth control using modern contraceptives					
Disagree	34 (28.8)	Ref		Ref	
Moderately agree	54 (36.5)	1.419 (0.844–2.388)	0.187	2.029 (1.036–3.976)	0.039
Agree	20 (57.1)	3.294 (1.512–7.179)	0.003	4.126 (1.501–11.342)	0.006
In our culture, men are the ones who decides when to have a child					
Disagree	25 (62.5)	4.279 (2.033–9.008)	< 0.001	5.968 (2.346–15.181)	< 0.001
Moderately agree	46 (35.7)	1.423 (0.843–2.402)	0.187	2.044 (1.048–3.986)	0.036
Agree	37 (28.0)	Ref		Ref	
Wives need husbands’ encouragement of modern contraceptive use					
Disagree	16 (59.3)	3.732 (1.626–8.566)	0.002	7.029 (2.574–19.194)	< 0.001
Moderately agree	39 (45.9)	2.176 (1.278–3.703)	0.004	2.316 (1.186–4.522)	0.014
Agree	53 (28.0)	Ref		Ref	
Men are involved in programs that seek to address women’s uptake of contraception					
Strongly disagree	8 (22.9)	Ref		Ref	
Disagree	17 (22.7)	0.989 (0.380–2.574)	0.982	1.094 (0.317–3.771)	0.887
Moderately agree	34 (33.0)	1.663 (0.683–4.047)	0.262	2.851 (0.894–9.093)	0.077
Agree	40 (54.8)	4.091 (1.641–10.201)	0.003	7.765 (2.299–26.226)	0.001
Strongly agree	9 (60.0)	5.062 (1.380–18.572)	0.014	5.423 (1.136–25.884)	0.034

To enhance interpretability, the AORs were comparatively summarized to highlight the strongest predictors of modern contraceptive use. Low religious influence (AOR = 13.918) and male involvement in reproductive decision‐making (AOR = 7.765) emerged as the most powerful determinants, followed by women′s empowerment (AOR = 4.322) and positive cultural orientation (AOR = 3.680). In contrast, traditional gender norms and strong religious restrictions were associated with significantly reduced likelihood of use. This comparative narrative presentation serves the same purpose as a forest plot by clarifying the direction and relative strength of effects without requiring an additional figure (Table 5).

Although most predictors were statistically significant (*p* < 0.05), it is important to interpret these results in terms of effect size and precision. The AORs indicate the magnitude of association, whereas the 95% CIs show the range of plausible values. For instance, women who reported minimal religious influence had an AOR of 13.918 (95% CI: 2.54–76.30), signifying a strong but variable effect. The wide CI suggests some uncertainty, possibly due to small subgroup sizes, implying that the practical impact may be smaller than the statistical value suggests. Similarly, male involvement in reproductive health programs (AOR = 7.765, 95% CI: 2.30–26.23) demonstrates both statistical and practical importance. These results highlight that cultural and religious engagement substantially shape contraceptive behavior, but the magnitude of these effects should be viewed with measured interpretation.

The presence of relatively large odds ratios for some predictors (e.g., AOR = 13.918 for reduced religious influence) may suggest model instability or small cell effects within certain categorical levels. To evaluate this, multicollinearity diagnostics (VIF < 2.0) and residual analyses were performed, indicating no major collinearity problems. Nonetheless, the model′s strength (Nagelkerke R^2^ = 0.392) implies moderate explanatory power, and some wide CIs reflect limited precision in smaller strata. Future studies employing larger or longitudinal datasets would allow more stable estimation of these relationships and could test for potential interaction effects between religion, gender norms, and education. Recognizing these limitations ensures that statistical significance is not overstated and that the observed associations are interpreted conservatively.

Support from religious leaders was identified as a major factor. Women who agreed that their religious leader openly supports family planning were more likely to use contraception (AOR = 7.085, *p* < 0.001), consistent with Adedini et al. [5], who underlined that endorsement by religious figures enhances contraceptive use. Similarly, women who believed their religion supported contraception were more likely to use modern techniques (AOR = 2.974, *p* = 0.023), which was comparable with the findings of Turner [2] in South Africa. Religious leaders have a considerable influence on societal ideals and communal actions, particularly in conservative cultures. When religious leaders publicly support family planning, they serve to legitimize contraception usage within a moral and cultural context that could otherwise prohibit it. Their support removes religious stigma, reassures followers that family planning is compatible with faith, and allows women to access contraceptive treatments without fear of being judged [10]. Similarly, when people believe their faith supports contraception, they are more confident and eager to use modern techniques. This internal religious approbation links reproductive decisions to spiritual ideals, making family planning a responsible and acceptable option [49]. In settings like Garbatula, where religious teachings frequently impact public sentiment, religious leaders′ positive framing of contraception is critical for increasing acceptance.

Cultural gender standards also had an important impact. Women who disagreed with men deciding when to have children were nearly six times more likely to use contraception (AOR = 5.968, *p* < 0.001), corroborating Negash et al. [50] findings that autonomy in decision‐making boosts contraceptive usage. Women who disagreed with husbands′ encouragement to use contraception were seven times more likely to use it (AOR = 7.029, *p* < 0.001), supporting the findings of Sarfraz et al. [41]. In traditional patriarchal countries, male authority over reproduction decisions frequently limits women′s access to and choice of contraception. When women reject the concept that males should make all parenting decisions, they gain more control over their reproductive choices, which leads to increased contraceptive use [7].

Similarly, when women believe they do not need their husband′s encouragement to use contraception, they have independent agency, allowing them to seek and embrace modern family planning methods without relying on male approval [51]. In such circumstances, women′s empowerment, whether through education, economic engagement, or access to reproductive health information, is critical in breaking down conventional gender stereotypes, fostering reproductive self‐determination, and encouraging increased use of modern contraception.

Interestingly, although male participation in reproductive health programs significantly increased contraceptive use (AOR = 7.765, *p* = 0.001 for those who agreed), the impact on those who only moderately agreed was weaker and not statistically significant (AOR = 2.851, *p* = 0.077), implying that active and visible male participation, rather than passive support, is critical for behavior change.

The findings indicate that active and visible male participation in reproductive health initiatives is critical for significant change in contraceptive practice. When males fully participate in teaching sessions, shared decision‐making, and public support, they help to normalize family planning in the community, dispel myths, and minimize gender‐based resistance, hence increasing contraceptive use [10]. In contrast, moderate or passive support, in which males agree in principle but do not actively participate, has little influence since it fails to challenge old norms or firmly empower women. Without solid male advocacy, women may continue to endure subtle pressures or cultural stigma, which can impede contraceptive adoption. As a result, the study emphasizes that the quality and depth of male engagement, rather than simple verbal agreement, are important determinants of improved reproductive health outcomes.

In summary, the analytical results remain robust across diagnostic checks and sensitivity analyses. Despite some variability in effect sizes, the direction and significance of relationships consistently underscore the pivotal role of religion, gender norms, and male engagement in influencing contraceptive uptake. In line with national demographic patterns, contraceptive use in this study was higher among younger, educated, and economically active women. These factors likely enhance access to reproductive information and negotiation power within households, thereby amplifying the positive influence of supportive religious and cultural environments. Additionally, a comparative summary of AORs provided a clear overview of predictor strength, demonstrating that religious moderation and male participation had the most substantial positive effects on contraceptive uptake.

## 4. Discussion and Contextualization

The purpose of this discussion is to interpret the study′s findings within broader theoretical, national, and regional contexts. Building on the results presented, the section explores how cultural, religious, and gender dynamics influence modern contraceptive use among married women in Garbatula sub‐county. Instead of replicating statistical results, the discussion analyzes the underlying social mechanisms that underpin these connections and places them in the context of previous research from Kenya and Africa. The section also highlights important methodological limitations, provides conclusions for county‐level reproductive health policy, and compares the study′s results with national data from the Kenya Demographic and Health Survey (KDHS). In doing so, it offers a coherent narrative that connects empirical findings to practical implications for enhancing family planning uptake in pastoralist and faith‐based communities.

### 4.1. Interpretation of Findings

The results of this study show that through interrelated social and interpersonal mechanisms, cultural and religious dynamics significantly influence contraceptive behavior. Women are able to reconcile the use of contraceptives with their spiritual views when religious constraints are lessened and supportive religious leaders are involved. This seems to reframe moral perceptions about family planning. Such religious support aids in changing the narrative so that contraception is seen as a responsible act of stewardship and family welfare rather than as a moral violation. This moral reframing is essential for normalizing conversations about reproductive health in places like Garbatula, where faith has a significant impact on day‐to‐day living. The influence of gender relations and male engagement on women′s reproductive decisions is equally significant. The fear of partner rejection that frequently discourages the use of contraceptives in pastoralist communities is lessened when males actively participate in decision‐making processes, enhancing women′s autonomy and shared responsibility. This dynamic demonstrates that the adoption of contraceptives is a result of negotiation within societal hierarchies, where gender standards, religious authority, and family power relations intersect, rather than just being a question of personal preference. Therefore, rather than focusing only on women, strengthening couple‐based and community‐level treatments that address these relationship elements can promote more long‐lasting behavior change.

### 4.2. Comparison With National and Regional Evidence

According to the KDHS [52], Garbatula′s recorded prevalence of contraception is in line with more general national trends. Approximately 57% of married women nationwide utilize a modern method of contraception; however, uptake is much lower in the desert, primarily Muslim counties like Mandera and Isiolo, where rates are below 35%. These tendencies are similar to the sociocultural dynamics found in Garbatula, where the use of contemporary contraceptives is restricted by patriarchal norms, faith‐based beliefs, and a lack of exposure to reproductive health education. This consistency implies that the obstacles found in this study are part of a larger structural issue that affects Kenya′s pastoralist and marginalized areas. Similar patterns have also been observed throughout sub‐Saharan Africa, especially in Ethiopia [17] and northern Nigeria [5], where male‐dominated household decision‐making and religious conservatism equally discourage the use of contraceptives. These cross‐national similarities draw attention to a recurrent theme: socioreligious norms and power dynamics, rather than service availability alone, significantly influence reproductive behavior in faith‐oriented countries. Therefore, culturally grounded approaches that involve religious and community leaders while addressing gender disparities that restrict women′s autonomy are necessary to address the continuance of limited contraceptive use in such situations.

Although similar studies have been conducted in Ethiopia and northern Kenya, the present research advances existing knowledge in two key ways. First, it quantifies the relative strength of cultural and religious predictors through logistic regression, offering precise estimates of their influence on contraceptive uptake, an approach rarely applied in pastoralist Muslim settings. Second, it contextualizes these quantitative findings within Garbatula′s unique socioreligious environment, where religious authority and gender dynamics intersect more visibly than in other Kenyan counties. These distinctions enhance the study′s theoretical and practical relevance by translating local experiences into measurable determinants that can inform targeted interventions.

### 4.3. Policy Implications

These results have significant practical implications for county‐level reproductive health planning, particularly in pastoralist and faith‐based environments like Isiolo county. Incorporating religious leaders into family planning activism might help eliminate long‐standing moral stigmas associated with contemporary technologies and normalize conversations about contraception within congregations. Spousal communication and shared decision‐making can also be improved by strengthening male‐centered reproductive health education through community forums, local radio programs, and peer advocacy groups. Additionally, by boosting autonomy, self‐efficacy, and exposure to accurate reproductive health information, women′s empowerment initiatives such as adult literacy programs, savings groups, and microenterprise projects can indirectly increase the uptake of contraceptives. These findings highlight the necessity of multisectoral approaches that incorporate gender and cultural factors into county planning frameworks from a policy standpoint. As key pillars of behavior change communication, faith‐sensitive and gender‐responsive techniques should be given priority in the County Integrated Development Plan (CIDP) and associated reproductive health policies. In order to guarantee that family planning treatments align with local values and belief systems, cooperation between the Ministry of Health, county health departments, and faith‐based organizations can foster long‐term community involvement. These culturally based strategies are more likely to increase long‐term program efficacy, decrease opposition, and promote community ownership.

### 4.4. Limitations

The methodological scope of this study is defined by a number of limitations that should be taken into consideration when interpreting its results. Because the data record associations at a single point in time rather than following changes over an extended period of time, the cross‐sectional approach restricts the ability to demonstrate causality. Therefore, longitudinal or mixed‐method research may offer an improved comprehension of how cultural and religious beliefs change over time and impact contraceptive practice. Furthermore, as all of the data were self‐reported, recall bias and social desirability bias might have been introduced, especially in a setting where conversations about contraception can be morally or socially sensitive. In order to comply with social or religious norms, respondents might have overstated or underreported their usage of contraceptives. The nomadic and difficult‐to‐reach Garbatula communities may still be underrepresented despite the study′s high response rate (93.8%), as their movement and scattered settlement patterns hindered regular contact during data collection. This could have a minor impact on the sample′s representativeness and the results′ applicability to the larger sub‐county population. Additionally, important variables like cultural and religious views were operationalized using category measures, which may have oversimplified the multifaceted character of belief systems and social norms despite their analytical value. To fully capture the complexity and complexity of these concepts, future research may use qualitative or ethnographic methods. In order to improve the validity, reliability, and interpretive credibility of the results, the study included a number of methodological safeguards, such as random sampling, model diagnostics (Hosmer–Lemeshow test, VIF checks), and contextual triangulation.

## 5. Conclusion and Recommendations

This study provides evidence that broadens the national conversation on faith and family planning by capturing contraceptive dynamics within pastoralist Muslim communities in Kenya, in contrast to the majority of earlier studies that concentrated on urban or mixed‐faith groups. The study concludes that modern contraceptive use among married women in Garbatula sub‐county is profoundly shaped by religious beliefs, cultural norms, and gender relations. Reduced religious restrictions, supportive faith leadership, and male involvement in reproductive decision‐making were the most influential enablers of contraceptive uptake. Conversely, patriarchal control and rigid religious interpretations constrained women′s autonomy and acceptance of family planning. These findings underscore that contraceptive behavior in faith‐based and pastoralist contexts is not merely a matter of individual choice but a reflection of broader social, moral, and relational dynamics.

To enhance contraceptive uptake, reproductive health interventions should adopt culturally responsive and faith‐sensitive approaches. County governments, through the Ministry of Health and community‐based organizations, should actively engage religious leaders as advocates for responsible parenthood and reproductive well‐being. Male‐focused education programs and women′s empowerment initiatives should be prioritized to strengthen joint decision‐making and autonomy. In addition, integrating these strategies within the CIDP will ensure that family planning programs are contextually grounded, inclusive, and sustainable. Finally, future research should employ longitudinal and qualitative designs to capture how evolving cultural and religious attitudes continue to shape contraceptive practices in pastoralist communities.

## Conflicts of Interest

The authors declare no conflicts of interest.

## Funding

No funding was received for this manuscript.

## Data Availability

The anonymized dataset and analysis syntax used in this study are available upon reasonable request to the corresponding author. Due to the sensitivity of reproductive health data and to protect participant confidentiality, the dataset is not publicly archived but can be shared with qualified researchers under a data‐use agreement.

## References

[1] Ullah M. A. , Kamal S. M. M. , Begum R. , and Wahid T. , Women Health, Empowerment and its Association With Contraceptive Use, Journal of Angiotherapy. (2024) 8, no. 6, 1–12, 10.25163/angiotherapy.869768.

[2] Turner N. , Influence of Religion and Religiosity on Fertility and Contraceptive Use in Continental Sub-Saharan Africa: A Comprehensive Review , 2021, 10.31237/osf.io/sezdq.

[3] Burfoot A. and Güngör D. , Women and Reproductive Technologies: The Socio-Economic Development of Technologies Changing the World, 2022, Taylor & Francis., 10.4324/9780429467646.

[4] Rich S. , Haintz G. L. , McKenzie H. , and Graham M. , Factors that Shape Women’s Reproductive Decision-Making: A Scoping Review, Journal of Research in Gender Studies. (2021) 11, no. 2, 9–31, 10.22381/JRGS11220211.

[5] Adedini S. A. , Babalola S. , Ibeawuchi C. , Omotoso O. , Akiode A. , and Odeku M. , Role of Religious Leaders in Promoting Contraceptive Use in Nigeria: Evidence From the Nigerian Urban Reproductive Health Initiative, Global Health: Science and Practice. (2018) 6, no. 3, 500–514, 10.9745/GHSP-D-18-00135, 2-s2.0-85054449378, 30287529. 30287529 PMC6172128

[6] Cole W. M. and Geist C. , Conceiving of Contraception: World Society, Cultural Resistance, and Contraceptive Use, 1970–2012, Social Forces. (2021) 99, no. 4, 1394–1431, 10.1093/sf/soaa077.

[7] D’Souza P. , Bailey J. V. , Stephenson J. , and Oliver S. , Factors Influencing Contraception Choice and Use Globally: A Synthesis of Systematic Reviews, European Journal of Contraception and Reproductive Health Care. (2022) 27, no. 5, 364–372, 10.1080/13625187.2022.2096215, 36047713. 36047713

[8] Kriel Y. , Milford C. , Cordero J. P. , Suleman F. , Steyn P. S. , and Smit J. A. , Access to Public Sector Family Planning Services and Modern Contraceptive Methods in South Africa: A Qualitative Evaluation From Community and Health Care Provider Perspectives, PLoS One. (2023) 18, no. 3, e0282996, 10.1371/journal.pone.0282996, 36930610. 36930610 PMC10022780

[9] Bashir N. M. , Beyond the Health Care Setting: Exploring the Intersections of Gender, Culture and Religion and Their Influence on Utilization of Family Planning Services in Northern, 2022, Lancaster University (United Kingdom), https://eprints.lancs.ac.uk/id/eprint/169925.

[10] Kabagenyi A. , Reid A. , Ntozi J. , and Atuyambe L. , Socio-Cultural Inhibitors to Use of Modern Contraceptive Techniques in Rural Uganda: A Qualitative Study, Pan African Medical Journal. (2016) 25, 1–12, 10.11604/PAMJ.2016.25.78.6613, 2-s2.0-85016446092. PMC5324155 28292041

[11] Birch J. and Heyes C. , The Cultural Evolution of Cultural Evolution, Philosophical Transactions of the Royal Society B: Biological Sciences. (2021) 376, no. 1828, 10.1098/rstb.2020.0051, 33993760. PMC8126465 33993760

[12] Arousell J. , Carlbom A. , Johnsdotter S. , and Essén B. , Are ‘Low Socioeconomic Status’ and ‘Religiousness’ Barriers to Minority Women’s Use of Contraception? A Qualitative Exploration and Critique of a Common Argument in Reproductive Health Research, Midwifery. (2019) 75, 59–65, 10.1016/j.midw.2019.03.017, 2-s2.0-85064430894. 31005014

[13] Sinai I. , Omoluabi E. , Jimoh A. , and Jurczynska K. , Unmet Need for Family Planning and Barriers to Contraceptive Use in Kaduna, Nigeria: Culture, Myths and Perceptions, Culture, Health & Sexuality. (2020) 22, no. 11, 1253–1268, 10.1080/13691058.2019.1672894, 31662042. 31662042

[14] Sortland T. E. , Samburu Youth Navigating Violent Terrains. Reconfiguring Samburu Masculinity in Northern Kenya, 2017, https://hdl.handle.net/1956/16125.

[15] Stavitz J. , Understanding Micronutrient Access Through the Lens of the Social Ecological Model: Exploring the Influence of Socioeconomic Factors—A Qualitative Exploration, Nutrients. (2024) 16, no. 11, 10.3390/nu16111757, 38892693. PMC11174576 38892693

[16] Bagaja H. G. , Factors Influencing Utilization of Free Maternity Care Services Among Pastoralist Women in Isiolo County, 2024, Maseno University, https://repository.maseno.ac.ke/handle/123456789/6297.

[17] Tekelab T. , Melka A. S. , and Wirtu D. , Predictors of Modern Contraceptive Methods Use Among Married Women of Reproductive Age Groups in Western Ethiopia: A Community Based Cross-Sectional Study, BMC Women’s Health. (2015) 15, no. 1, 1–8, 10.1186/s12905-015-0208-z, 2-s2.0-84937213702, 26183090. 26183090 PMC4504461

[18] Alvergne A. and Stevens R. , Cultural Change Beyond Adoption Dynamics: Evolutionary Approaches to the Discontinuation of Contraception, Evolutionary Human Sciences. (2021) 3, 10.1017/ehs.2021.8, 37588536. PMC10427300 37588536

[19] Dempsey R. C. , McAlaney J. , and Bewick B. M. , A Critical Appraisal of the Social Norms Approach as an Interventional Strategy for Health-Related Behavior and Attitude Change, Frontiers in Psychology. (2018) 9, 1–16, 10.3389/fpsyg.2018.02180, 2-s2.0-85055972110, 30459694. 30459694 PMC6232455

[20] Hall K. S. , Manu A. , Morhe E. , Dalton V. K. , Challa S. , Loll D. , Dozier J. L. , Zochowski M. K. , Boakye A. , and Harris L. H. , *Bad Girl* And Unmet Family Planning Need Among Sub-Saharan African Adolescents: The Role of Sexual And Reproductive Health Stigma, Qualitative Research in Medicine & Healthcare. (2018) 2, no. 1, 55–64, 10.4081/qrmh.2018.7062. 30556052 PMC6292434

[21] Lingenfelter S. , Yap: Political Leadership and Culture Change in an Island Society, 2019, University of Hawaii Press, https://books.google.co.ke/books?hl=en%26lr=%26id=AVrGDwAAQBAJ%26oi=fnd%26pg=PR1.

[22] Mochache V. , Wanje G. , Nyagah L. , Lakhani A. , El-Busaidy H. , Temmerman M. , and Gichangi P. , Religious, Socio-Cultural Norms and Gender Stereotypes Influence Uptake and Utilization of Maternal Health Services Among the Digo Community in Kwale, Kenya: A Qualitative Study, Reproductive Health. (2020) 17, no. 1, 1–10, 10.1186/s12978-020-00919-6, 32448327. 32448327 PMC7245746

[23] Rahman M. M. , Mostofa M. G. , and Hoque M. A. , Women’s Household Decision-Making Autonomy and Contraceptive Behavior Among Bangladeshi Women, Sexual & Reproductive Healthcare. (2014) 5, no. 1, 9–15, 10.1016/j.srhc.2013.12.003, 2-s2.0-84892973623, 24472384. 24472384

[24] Rossi P. , Strategic Choices in Polygamous Households: Theory and Evidence From Senegal, Review of Economic Studies. (2019) 86, no. 3, 1332–1370, 10.1093/restud/rdy052, 2-s2.0-85072370853.

[25] Agbadi P. , Eunice T. T. , Akosua A. F. , and Owusu S. , Complex Samples Logistic Regression Analysis of Predictors of the Current Use of Modern Contraceptive Among Married or in-Union Women in Sierra Leone: Insight From the 2013 Demographic and Health Survey, PLoS One. (2020) 15, no. 4, 40–44, 10.1371/journal.pone.0231630, 32298333. PMC7162447 32298333

[26] Saoke V. O. , Musafiri C. M. , Ndwiga Z. N. , and Githaiga P. W. , The Christian Religious Education Teachers ’ Attitudes Toward the Five-Stage Lesson Plan Framework in Kenya : A Gender-Based Analysis, Heliyon. (2023) 9, no. 8, e19104, 10.1016/j.heliyon.2023.e19104, 37636406. 37636406 PMC10448468

[27] Saoke V. O. , Ndwiga Z. N. , Githaiga P. W. , Gitonga C. M. , Kubai K. I. , Nzomo C. M. , Mulonzi B. M. , and Ngicho D. O. , The Relationship Between Teachers’ Planning and Content Delivery Using the Five-Stage Lesson Plan Structure: An Analysis of Age, Gender, Experience and Academic Qualifications in Kenya, European Journal of Education. (2025) 60, no. 1, 1–13, 10.1111/ejed.70021.

[28] Saoke V. O. , Ndwiga Z. N. , Githaiga P. W. , and Musafiri C. M. , Determinants of Awareness and Implementation of Five-Stage Lesson Plan Framework Among Christian Religious Education Teachers in Meru County, Heliyon. (2022) 8, 10.1016/j.heliyon.2022.e11177. PMC9634273 36339759

[29] Handa S. , Prasad S. , Rajashekharappa C. B. , Garg A. , Ryana H. K. , and Khurana C. , Oral Health Status of Rural and Urban Population of Gurgaon Block, Gurgaon District Using WHO Assessment Form Through Multistage Sampling Technique, Journal of Clinical and Diagnostic Research. (2016) 10, no. 5, ZC43–ZC451, 10.7860/JCDR/2016/19048.7756, 2-s2.0-84965137011, 27437359. PMC4948535 27437359

[30] Daraz U. , Ullah H. , and Mulk J. U. , Illuminating the Path: Unleashing the Power of Education for Women’s Empowerment in Health, Journal of Positive School Psychology. (2023) 2023, no. 6, 889–902, https://journalppw.com/index.php/jpsp/article/view/17238.

[31] Woolson R. F. , Bean J. A. , and Rojas P. B. , Sample Size for Case-Control Studies Using Cochran’s Statistic, Biometrics. (1986) 927–932, 10.2307/2530706, 2-s2.0-0022964981. 3814733

[32] World Medical Association , Declaration of Helsinki: Ethical Principles for Medical Research Involving Human Subjects, 2013, https://www.wma.net/policies-post/wma-declaration-of-helsinki-ethical-principles-for-medical-research-involving-human-subjects/. 10.1001/jama.2013.281053 24141714

[33] Council for International Organizations of Medical Sciences (CIOMS), & World Health Organization , International Ethical Guidelines for Health-Related Research Involving Humans, 2016, 4th edition., CIOMS, Geneva, Retrieved from https://www.ncbi.nlm.nih.gov/books/NBK614410/. 40523065

[34] Abdulqader Q. M. , Applying the Binary Logistic Regression Analysis on the Medical Data, Science Journal of University of Zakho. (2017) 5, no. 4, 330–334, 10.25271/2017.5.4.388.

[35] Chen M. Y. , Lai L. J. , Chen H. C. , and Gaete J. , Development and Validation of the Short-Form Adolescent Health Promotion Scale, BMC Public Health. (2014) 14, no. 1, 10.1186/1471-2458-14-1106, 2-s2.0-84938066422, 29871621. PMC4216378 25344693

[36] Riegel B. , Dickson V. V. , and Vellone E. , The Situation-Specific Theory of Heart Failure Self-Care: An Update on the Problem, Person, and Environmental Factors Influencing Heart Failure Self-Care, Journal of Cardiovascular Nursing. (2022) 37, no. 6, 515–529, 10.1097/JCN.0000000000000919. 35482335 PMC9561231

[37] Arousell J. and Carlbom A. , Culture and Religious Beliefs in Relation to Reproductive Health, Best Practice and Research: Clinical Obstetrics and Gynaecology. (2016) 32, 77–87, 10.1016/j.bpobgyn.2015.08.011, 2-s2.0-84961202218. 26542927

[38] Afrilsah M. , Investigating the Role of Religion in Shaping Moral Values and Social Norms, Mahogany Journal De Social. (2024) 1, no. 1, 29–36, 10.37899/mjds.v1i1.7.

[39] Tigabu S. , Demelew T. , Seid A. , Sime B. , and Manyazewal T. , Socioeconomic and Religious Differentials in Contraceptive Uptake in Western Ethiopia: A Mixed-Methods Phenomenological Study, BMC Women’s Health. (2018) 18, no. 1, 1–10, 10.1186/s12905-018-0580-6, 2-s2.0-85048144432, 29871621. 29871621 PMC5989360

[40] Anwar L. , Asghar O. I. , and Ejaz K. , Role of Working Women in Decision Making About Family Matters in District Poonch Azad Jammu and Kashmir, Harf-O-Sukhan. (2021) 5, no. 3, 497–509, https://www.harf-o-sukhan.com/index.php/Harf-o-sukhan/article/view/761.

[41] Sarfraz M. , Hamid S. , Kulane A. , and Jayasuriya R. , ‘The Wife Should Do as Her Husband Advises’: Understanding Factors Influencing Contraceptive Use Decision Making Among Married Pakistani Couples—Qualitative Study, PLoS One. (2023) 18, no. 2, 1–19, 10.1371/journal.pone.0277173, 36795781. PMC9934449 36795781

[42] Shultz J. M. , Cooper J. L. , Baingana F. , Oquendo M. A. , Espinel Z. , Althouse B. M. , Marcelin L. H. , Towers S. , Espinola M. , McCoy C. B. , Mazurik L. , Wainberg M. L. , Neria Y. , and Rechkemmer A. , The Role of Fear-Related Behaviors in the 2013–2016 West Africa Ebola Virus Disease Outbreak, Current Psychiatry Reports. (2016) 18, no. 11, 104–114, 10.1007/s11920-016-0741-y, 2-s2.0-84991387155, 27739026. 27739026 PMC5241909

[43] Onarheim K. H. , Iversen J. H. , and Bloom D. E. , Economic Benefits of Investing in Women’s Health: A Systematic Review, PLoS One. (2016) 11, no. 3, 1–23, 10.1371/journal.pone.0150120, 2-s2.0-84977628237, 27028199. PMC4814064 27028199

[44] Tilahun T. , Coene G. , Temmerman M. , and Degomme O. , Couple Based Family Planning Education: Changes in Male Involvement and Contraceptive Use Among Married Couples in Jimma Zone, Ethiopia, Ethiopia. BMC Public Health. (2015) 15, no. 1, 1–8, 10.1186/s12889-015-2057-y, 2-s2.0-84937203805, 26194476. PMC4509724 26194476

[45] Vouking M. Z. , Evina C. D. , and Tadenfok C. N. , Male Involvement in Family Planning Decision Making in Sub-Saharan Africa- What the Evidence Suggests, Pan African Medical Journal. (2014) 19, 1–5, 10.11604/pamj.2014.19.349.5090, 2-s2.0-84914110941, 25922638. 25922638 PMC4406389

[46] John N. A. , Babalola S. , and Chipeta E. , Sexual Pleasure, Partner Dynamics and Contraceptive Use in Malawi, International Perspectives on Sexual and Reproductive Health. (2015) 41, no. 2, 99–107, 10.1363/4109915, 2-s2.0-84938375362, 26308262. 26308262

[47] Ahmed F. , Malik N. I. , Bashir S. , Noureen N. , Ullah S. , Ahmed J. B. , Mansoor T. , and Tang K. , “An Obedient Wife Never Says ‘No’ to Her Virtual God.” High Fertility Conceptions and Barriers to Contraceptive Use Among Mothers of Southern Pakistan: A Qualitative Study, BMC Public Health. (2024) 24, no. 1, 10.1186/s12889-024-19484-9, 39118088. PMC11308514 39118088

[48] Muhamad Tri Utama A. , “You’re Planning a Family, Not Just a Pregnancy”: The Meanings, Experiences, and Uneven Burdens of Family Planning in Women’s Lives, 2022, 9, PhD diss., University of Delaware, https://udspace.udel.edu/handle/19716/33260.

[49] Marchin A. , Seale R. , Sheeder J. , Teal S. , and Guiahi M. , Integration of Catholic Values and Professional Obligations in the Provision of Family Planning Services: A Qualitative Study, JAMA Network Open. (2020) 3, no. 10, 10.1001/jamanetworkopen.2020.20297, 33044549. PMC7550969 33044549

[50] Negash W. D. , Eshetu H. B. , and Asmamaw D. B. , Intention to Use Contraceptives and its Correlates Among Reproductive Age Women in Selected High Fertility Sub-Saharan Africa Countries: A Multilevel Mixed Effects Analysis, BMC Public Health. (2023) 23, no. 1, 1–10, 10.1186/s12889-023-15187-9, 36747157. 36747157 PMC9901088

[51] Ewerling F. , Victora C. G. , Raj A. , Coll C. V. N. , Hellwig F. , and Barros A. J. D. , Demand for Family Planning Satisfied With Modern Methods Among Sexually Active Women in Low- and Middle-Income Countries: Who Is Lagging Behind?, Reproductive Health. (2018) 15, no. 1, 1–10, 10.1186/s12978-018-0483-x, 2-s2.0-85043283984, 29510682. 29510682 PMC5840731

[52] Kenya National Bureau of Statistics , Kenya Demographic and Health Survey, 2022, https://www.knbs.or.ke/reports/kdhs-2022/.

